# Supplementary figures and images for: Factors affecting feeding 6–23 months age children according to minimum acceptable diet in Ethiopia: A multilevel analysis of the Ethiopian Demographic Health Survey
Source: PLoS One. 2019 Feb 21;14(2):e0203098. doi: 10.1371/journal.pone.0203098 (PMC6383941; doi:10.1371/journal.pone.0203098)

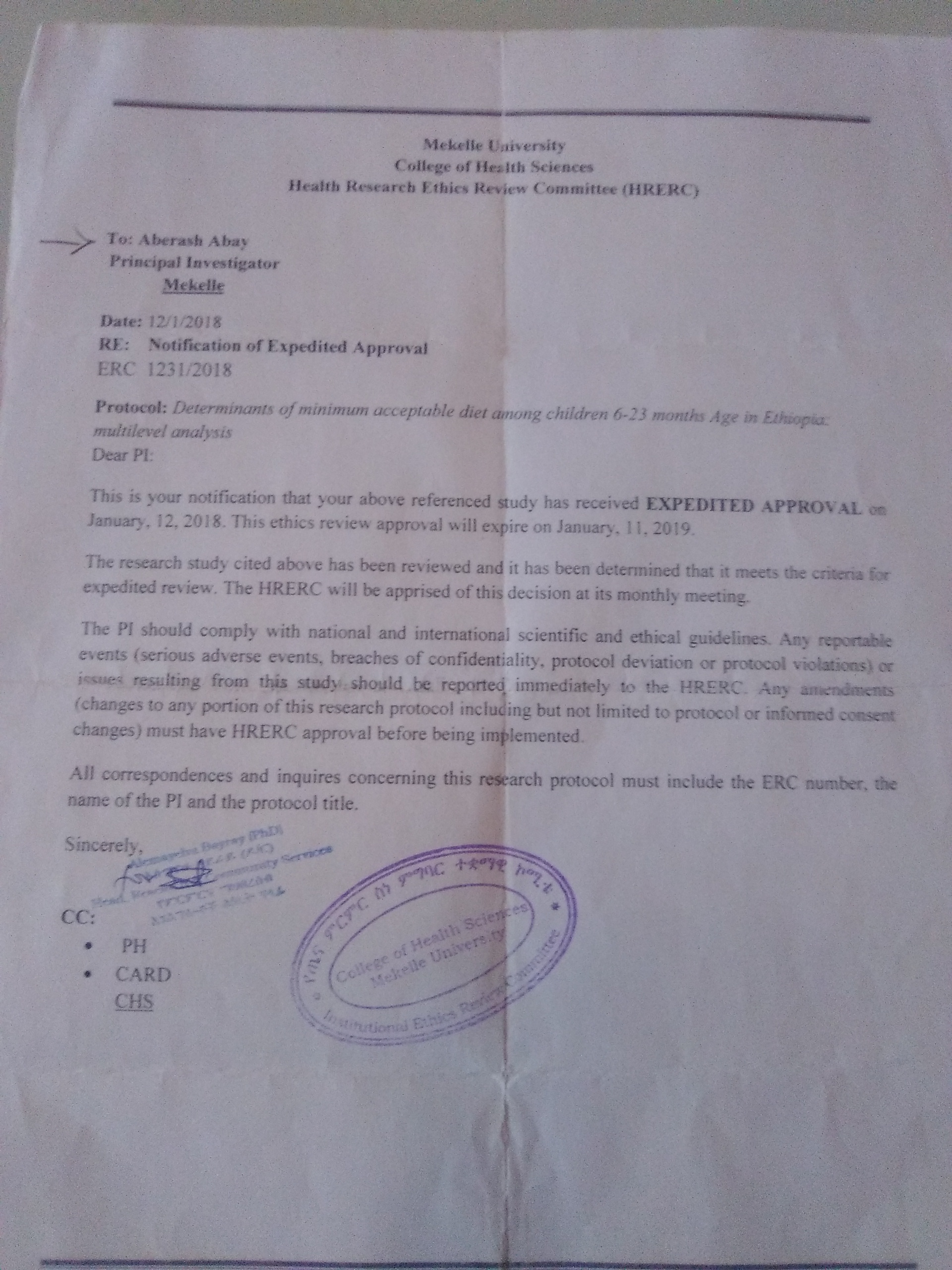

Supplement: S1 File — (JPG) [file pone.0203098.s001.jpg]

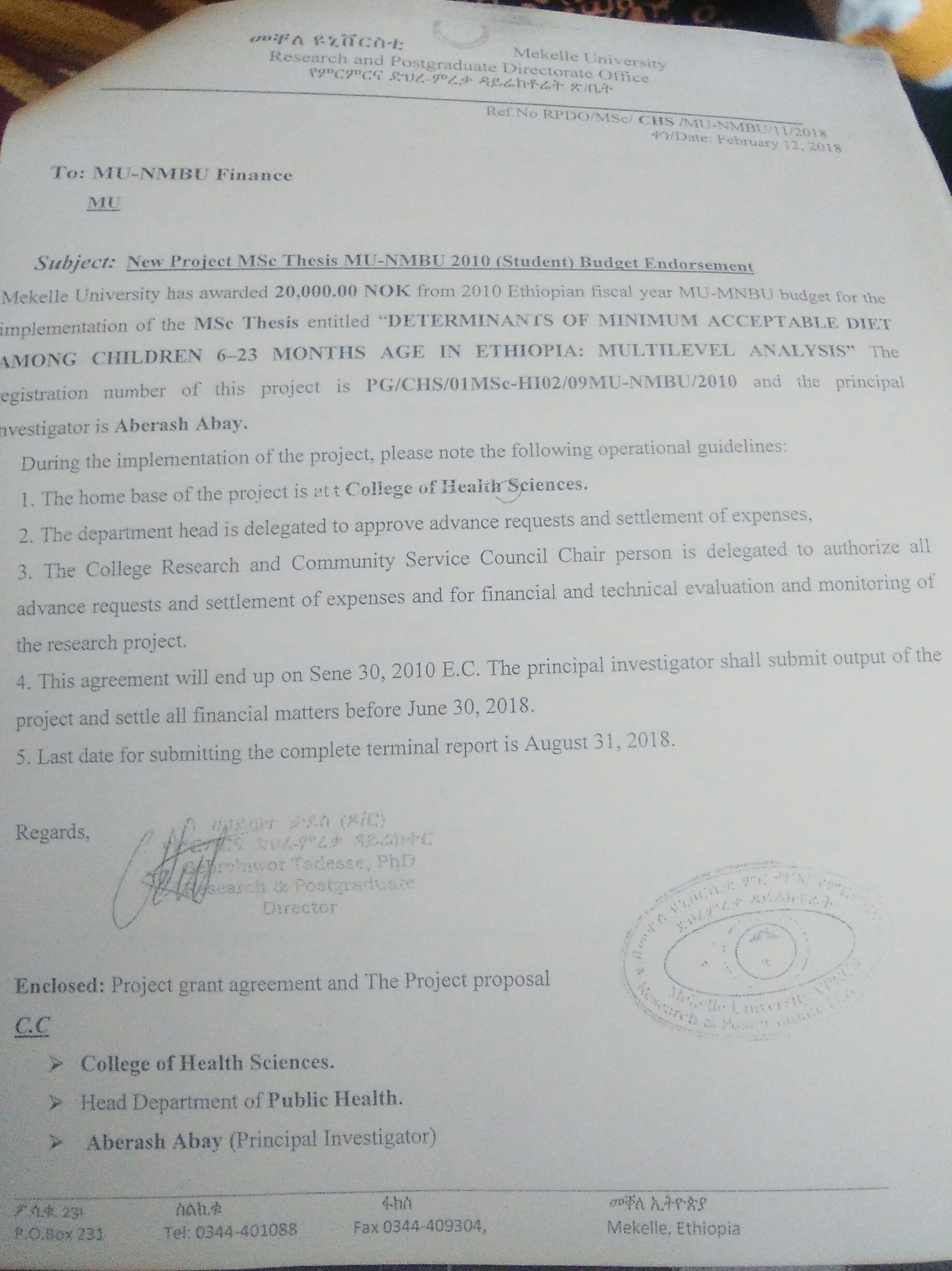

Supplement: S2 File — (JPG) [file pone.0203098.s002.jpg]
